# Pubertal timing: A life course pathway linking early life risk to adulthood cardiometabolic health

**DOI:** 10.1371/journal.pone.0299433

**Published:** 2024-03-27

**Authors:** Maria E. Bleil, Bradley M. Appelhans, Steven E. Gregorich, Robert A. Hiatt, Glenn I. Roisman, Cathryn Booth-LaForce

**Affiliations:** 1 Child, Family, & Population Health Nursing, University of Washington, Seattle, WA, United States of America; 2 Department of Preventive Medicine, Rush University Medical Center, Chicago, IL, United States of America; 3 Department of Medicine, University of California San Francisco, San Francisco, CA, United States of America; 4 Department of Epidemiology & Biostatistics, University of California, San Francisco, San Francisco, CA, United States of America; 5 Institute of Child Development, University of Minnesota, Minneapolis, MN, United States of America; Universitair Kinderziekenhuis Koningin Fabiola: Hopital Universitaire des Enfants Reine Fabiola, BELGIUM

## Abstract

**Objective:**

To evaluate a series of prospective life course models testing whether the timing of pubertal development is a pathway through which prepubertal risk factors may influence adulthood cardiometabolic health.

**Methods:**

Subjects were 655 female participants in the NICHD Study of Early Child Care and Youth Development (SECCYD) and recent SECCYD 30-year follow-up, the Study of Health in Early and Adult Life (SHINE). Prepubertal risk factors included maternal menarcheal age, child race/ethnicity, child health status indicators, and child adversity indicators. Pubertal timing was indexed by breast development onset (Tanner stage [TS] II), pubic hair onset (TS II) and menarcheal age. Adulthood cardiometabolic risk (CMR) was indexed by a composite of waist circumference, systolic blood pressure, diastolic blood pressure, hemoglobin A1c, C-reactive protein, and high-density lipoprotein.

**Results:**

Inspection of paths between the prepubertal risk factors, pubertal timing indicators, and adulthood CMR composite showed later breast development onset (-0.173, *p* < .01), later pubic hair onset (-0.182, *p* < .01), and later menarche (-0.145, *p* < .01) each predicted lower adulthood CMR, and each pubertal timing indicator mediated effects of prepubertal risk factors on adulthood CMR. Specifically, the timing of breast development onset and menarche mediated effects of maternal menarcheal age, Black (vs. White), Asian/PI (vs. White), child BMI percentile, and child SES on adulthood CMR (all *p*s < .05), and the timing of pubic hair onset mediated effects of maternal menarcheal age, Black (vs. White), and child BMI percentile on adulthood CMR (all *p*s < .10).

**Conclusion:**

Findings in the current study contribute to the broader literature by identifying pubertal development and its timing as a potentially important pathway through which early life exposures may shape adulthood cardiometabolic health and disease. These findings have important implications for novel opportunities for increased surveillance and potential intervention focusing on pubertal development as a target to improve health more broadly.

## Introduction

A growing body of evidence links adversity exposures in childhood to poor health in adulthood, generating intense interest in identifying the mechanisms that explain this association [1–3]. In this context, notable conceptual models have emerged describing adversity-related changes in biological systems that are hypothesized to be adaptive in responding to stress in the short-term but pose risks to the long-term maintenance of health [4–8]. These models, however, have not considered the role pubertal development and its unique physiological underpinnings may play. Fully understanding life course linkages between childhood adversity and adulthood health necessitates examination of the intervening developmental period—i.e., puberty—which notably is itself sensitive to the prepubertal environment and relates prospectively to myriad health and disease risk parameters in adulthood [9].

Pubertal development entails a set of biological processes leading to sexual maturation and the potential for human reproduction. In girls, gonadarche reflects the reactivation of the hypothalamic-pituitary–gonadal axis, stimulating ovarian follicle maturation and the synthesis of sex steroids that cause physical changes related to breast development, menarche, and ovulation [10]. Adrenarche, an independent process, reflects the maturation of the adrenal glands with increases in androgen production from the adrenal cortex causing physical changes related to axillary and pubic hair growth, body odor, and oily skin [10]. Markers of gonadarche and adrenarche are used to index the timing of pubertal development, which is itself highly variable [11]. Pubertal timing is commonly assessed by Tanner staging, a validated system in which separate dimensions of breast development (marking gonadarche) and pubic hair growth (marking adrenarche) are rated between stage 1 (pre-puberty) and stage 5 (full maturity) [12]. In addition, menarcheal age is commonly assessed by self- or mother-report. Menarche most typically occurs in Tanner stage IV breast development [13] and correlates with the onset of puberty (*r* = 0.53) [14]. Its assessment, although provided by self-report and often retrospective, has been shown to be highly reliable [15].

Inspection of these indicators reveals there are patterns of association that inform the timing of puberty. Predictors of earlier pubertal onset include younger maternal menarcheal age [16], reflecting factors shared intergenerationally, as well as minority racial/ethnic status, reflecting accelerated development among Black and Latina girls compared to their White counterparts [17–20]. In addition, markers of prepubertal body mass, including infancy weight gain, BMI percentile trajectories, and childhood obesity status have all been linked prospectively to earlier pubertal onset [21–23]. Beyond these established risk factors, a range of adverse experiences in early life have also been identified as predictors. In longitudinal studies, girls who experienced family-based hardships related to parenting quality, parent-child relationships, and stressful life events exhibited earlier pubertal onset and younger menarcheal age [14,24,25]. A similar pattern was observed among girls from families experiencing socioeconomic disadvantage [25–29].

Identifying factors that contribute to the timing of puberty has important clinical implications as earlier pubertal onset is related prospectively to a host of maladaptive outcomes. Earlier developing girls are at risk for depression and self-harm, disordered eating behaviors and poor self-image, as well as poor performance in school, substance use, teenage pregnancy, and sexually transmitted infections [30–35]. Additional risks to their physical health are evident in the short and long term. Earlier developing girls experience the early emergence and worsening of cardiovascular risk factors over time, including weight gain and increases in insulin, glucose, and blood pressure [36,37]. Associations extend into adulthood with increases in risk observed for obesity, type 2 diabetes, incident cardiovascular disease, cancer, and early mortality [38–43].

Why puberty, when initiated earlier versus later, imposes such risk across domains of psychosocial functioning and health remains poorly understood. Studies examining this question offer a number of hypotheses and speculation. One hypothesis suggests that when puberty is early or accelerated, physical changes misalign with the psychological maturity that is needed to adapt, leading to disruptions in functioning [44,45]. Although speculative, such disruptions likely impact patterns of sleep, activity, and diet relevant to later health. Another hypothesis suggests adverse environments signal the hastening of sexual maturation to prioritize the potential for reproduction despite other costs [46,47]. Finally, other explanations involve the application of conceptual models of risk [48–50]. Prominent prepubertal risk factors such as obesity, for example, may both trigger and further compound risk in interaction with concomitant hormonal and metabolic changes that occur normatively during puberty (e.g., reduced insulin sensitivity, increased androgens) [51,52], furthering risk into the post-pubertal period.

Building on this literature, in the current study, a series of prospective life course models were estimated to test links between prepubertal risk factors, pubertal timing, and adulthood cardiometabolic risk. The effects of prepubertal risk factors (i.e., child health and adversity exposures) on cardiometabolic risk, both direct and indirect, via pubertal timing, were modeled to test the hypothesis that the timing of pubertal development is a pathway through which early life exposures influence adulthood cardiometabolic health.

## Methods

### Participants

Subjects in the current study were participants in the NICHD SECCYD for which there was a recent 30-year follow-up, rebranded SHINE. The NICHD SECCYD (1991–2009) was a prospective study of children and their families followed between birth and adolescence to examine trajectories of child health and development [53]. The more recent SHINE (2018–2022) entailed a single, in-person follow-up visit among participants ages 26 to 31 years to characterize a breadth of social, behavioral, and health status information in adulthood [54]. See details in Bleil et al., 2023 [54] regarding the SHINE goals, methods, and measures.

NICHD SECCYD recruitment took place in 10 geographically diverse study sites in the United States: Seattle, WA; Madison, WI; Irvine, CA; Pittsburgh, PA; Wellesley, MA; Little Rock, AR; Philadelphia, PA; Morganton, NC; Lawrence, KS; and Charlottesville, VA. In the first 11 months of 1991, all mother-infant dyads of babies born within preselected 24-hour intervals at participating hospitals were screened. NICHD SECCYD exclusions were 1) mother >1 hour from the study site; 2) child being placed for adoption; 3) concurrent participation in another study; and 4) refusal to participate in initial screening. Additional sampling requirements (e.g., 10% single parent households) were imposed to ensure the sociodemographic composition of the final sample (N = 1364 families; n = 659 girls and n = 705 boys) represented the indicated geographies, based on the 1990 US Census. SHINE recruitment retained the original 10 study sites, but augmented recruitment efforts to reach participants who had moved away from the original sites by setting up ancillary sites, paying for participant travel to main or ancillary sites, and creating alternative study protocols that could be completed remotely. SHINE exclusions were 1) lack of prior written agreement to be re-contacted for future studies (ascertained at the end of the NICHD SECCYD); 2) current pregnancy / breastfeeding (temporary); and 3) current / recent cold or flu symptoms (temporary). On-going rescreening was conducted to monitor changes in temporary exclusions. Participant recruitment occurred between January 1st, 2018 and December 31st, 2022. NICHD SECCYD informed written consent and assent were obtained from parents and children, respectively. SHINE informed written consent was obtained from the now adult target participants. For the NICHD SECCYD, study approval was granted by the institutional review boards of each of the 10 university-based study sites, and, for SHINE, study approval was granted by the Human Subjects Division of the University of Washington.

The sample included the female participants in the NICHD SECCYD (n = 659). However, 4 participants who no longer identified as female (2 transgender male and 2 non-binary) were excluded, leaving n = 655 participants in the final analytic sample. Of these participants, 464 were eligible for SHINE because they agreed to be re-contacted for future studies following the conclusion of the NICHD SECCYD, and, of these participants, 374 (80.6%) participated in SHINE.

### Measures

#### Prepubertal risk factors

Background. Prepubertal background factors included maternal menarcheal age and child race/ethnicity. Maternal menarcheal age (in years) was assessed by self-reports of mothers queried at three different assessments from which the mean was computed. Child race/ethnicity was assessed by mother reports coded in five categories: Black, Latina, Asian/PI, ‘other’ race, and White.

Child health. Prepubertal child health was indexed by mother rated child ‘general’ health and child BMI percentile. Child general health was rated by mothers at child age 54 months in categories (1 = poor to 4 = excellent). Pre-pubertal BMI (weight in kg/height in m^2^) was derived using measurements of height and weight at child age 54 months. BMI percentile was then calculated using the 2000 CDC BMI-for-age clinical growth charts for girls.

Child adversity. *Prepubertal child SES* was indexed by parental education and family income-to-needs ratio. Educational attainment of the mother and father/partner at child age 1 month was assessed by self-report in six categories: 1 = less than high school; 2 = high school or general education diploma; 3 = some college or vocational degree; 4 = college degree; 5 = some graduate school or master’s degree; 6 = graduate degree greater than a master’s degree. A single parental education value was calculated by taking the mean of mother and father/partner educational attainment. Family income-to-needs ratio was assessed by self-report of family income and family size at child ages 1, 6, 15, 24, 36, and 54 months. A single value of family income-to-needs was calculated by taking the mean across ages. The sample distributions of the parental education and family income-to-needs ratio variables were standardized, then summed, and the result re-standardized to create a single prepubertal child SES composite with a sample mean of 0 and a standard deviation of 1. Higher composite scores reflected higher prepubertal child SES. *Prepubertal mother-child attachment* was assessed by the Strange Situation Procedure (SSP) at age 15 months, the Attachment Q-Sort (AQS) at age 24 months, and the Modified Strange Situation Procedure (MSSP) at age 36 months [55–57]. The proportion of times the child was coded secure (vs. insecure) across all assessments (ages 15, 24, and 36 months) was computed to produce a composite of prepubertal mother–child attachment [58]. Interrater agreement was 83% (к = .69) and 76% (к = .58) for the SSP and the MSSP, respectively, and the intraclass correlation was .96 for the AQS. *Prepubertal maternal sensitivity* was assessed using 15-minute semi-structured interactions between the mother and child at child ages 6, 15, 24, 36, and 54 months which were videotaped and rated. Ratings across all assessments were standardized and summed to produce a composite of prepubertal maternal sensitivity. Intercoder reliability was high at each assessment time point [59]. *Prepubertal negative life events* were assessed using the Life Experiences Survey (LES) [60] completed by the mother at child age 54 months. The LES entails a list of 57 life events, ranging from routine (e.g., start of school) to traumatic (e.g., death of a parent). The occurrence of each event (in past year) and its impact was rated. Items with a negative impact were summed and categories derived (0 = no events, 1 = 1–5 events, 2 = 6–10 events, 3 = 11+ events) to produce an ordinal composite of prepubertal negative life events. Adequate psychometrics have been reported [60].

#### Pubertal timing

Medical providers were trained and re-certified annually to conduct physical exams each year between child ages 9.5 and 15.5 years [11] from which sexual maturity was characterized using Tanner stage (TS) criteria [12,61], augmented by breast bud palpation. Photographs depicting typical development were used to compare and rate stages of sexual maturity for breast and pubic hair development, separately, ranging between TS I (pre-puberty) and TS V (full sexual maturity) [17]. Girls who were between stages were assigned to the earlier stage [11]. Annual evaluations continued until menarche and full sexual maturity (TS V) for breast and pubic hair development were reached. Menarcheal age was determined by querying the girls and their mothers. Mothers’ reports were used if girls’ reports were missing. Based on these exams, 3 indicators of pubertal onset were examined as the primary outcomes of interest: Age at the onset of breast development (TS II); 2) age at the onset of pubic hair development (TS II); and 3) menarcheal age.

#### Adulthood cardiometabolic risk (CMR) composite

The adulthood CMR composite included waist circumference (WC), systolic blood pressure (SBP), diastolic blood pressure (DBP), hemoglobin A1c (HbA1c), C-reactive protein (CRP), and high-density lipoprotein (HDL).

*WC*. WC was assessed using a tension-controlled tape measure positioned at the participant’s midpoint between the iliac crest and lowest rib. The measurement was taken on the exhalation and repeated until consecutive measurements were within 0.2 cm. The mean of these final two values was calculated to form the WC indicator.

*SBP and DBP*. SBP and DBP were assessed using a research grade, automated blood pressure monitor, pre-programmed to take three consecutive measurements with one-minute intervals between readings. The measurement was taken following a 5-minute rest period with the participant seated in a relaxed position and the cuff positioned on the left arm. The mean of these three values was calculated to form separate SBP and DBP indicators.

*HbA1c*, *CRP*, *and HDL*. Blood was drawn from the participant’s arm by a trained phlebotomist. Participants were pre-screened for cold and flu symptoms and rescheduled if symptomatic. The blood draw occurred between 7:00 and 10:00 am following an overnight fast and other timed restrictions (e.g., cessation of nicotine). Aliquots were frozen, and assays were performed in batches. HbA1c was assayed using a commercially available ELISA kit (E4656, ABcam, Walthon, MA); the inter-assay coefficient of variation (CV) was 10.4%, and the intra-assay CV was 8.1%. CRP was assayed using a commercially available ELISA kit (KHA0031, Invitrogen/Thermo Fisher Scientific, Waltham, MA); the inter-assay CV was 9.9%, and the intra-assay CV was 6.1%. HDL was assayed using conventional enzymatic methods.

After reverse scoring HDL, the sample distributions of all six cardiometabolic risk indicators (WC, SBP, DBP, HbA1c, CRP, and HDL) were standardized, then summed, and the result re-standardized to create a single CMR composite with a sample mean of 0 and a standard deviation of 1. Higher CMR composite scores reflected higher cardiometabolic risk.

### Analytical plan

Regression models were fit to examine links between prepubertal risk factors, pubertal timing indicators, and the adulthood CMR composite. First, the selected pubertal timing indicator was regressed onto the predictor variables, including the prepubertal ‘background’ variables (maternal menarcheal age, child race/ethnicity) and the prepubertal ‘child health’ variables (child general health, child BMI percentile), along with each prepubertal ‘adversity’ variable examined one-at-a-time (child SES, mother-child attachment, maternal sensitivity, negative life events). This process was repeated for breast development (TS II), pubic hair development (TS II), and menarcheal age, separately. Next, the adulthood CMR composite was regressed onto the same predictors noted above and, separately, each pubertal timing indicator. Finally, the mediating effects of the pubertal timing indicators were tested using a multi-step procedure. First, as an initial screen of each candidate indirect effect, the ‘joint’ test of significance [62] was assessed, i.e., both component paths—predictor to pubertal timing mediator as well as pubertal timing mediator to CMR composite—had *p*-values below an indicated alpha level which was set to be α = .10. Second, for indirect effects meeting the joint test criterion, exact *p*-values and CIs were then computed by partial posterior and hierarchical Bayesian methods [63], respectively. A multiple imputation model fit to data from the sample of *n* = 655 participants generated 30 completed data sets. The percentages of observed data points among variables included in the imputation model were as follows: 67.6% of all data points, 70.8% of SECCYD data points, and—among SHINE participants—83.2% of SHINE data points. Parameters and standard errors were estimated by combining results across imputed data sets [64,65].

## Results

### Descriptive analyses

In Table 1, descriptive statistics are reported for the main variables of interest. The racial/ethnic composition of the sample was 12.7% Black, 6.1% Latina, 1.7% Asian/PI, 3.3% ‘other’ race, and 76.2% White. Mean BMI percentile was 60.5 and 23.9% of girls were overweight or obese. For mothers and fathers/partners, 36.6% and 38.5%, respectively, received a college degree or higher with 13.6% of families reporting incomes below the federal poverty line. The mean age at breast development onset (TS II) was 9.8, the mean age at pubic hair onset (TS II) was 10.2, and the mean age at menarche was 12.4. With respect to the adulthood cardiometabolic risk indicators, 64.7% of women were above the risk threshold for waist circumference, 25.6% were considered hypertensive, 41.2% were prediabetic or diabetic, 16.2% had CRP values in the risk range, and 27.8% had HDL values in the risk range.

**Table 1:** Sample descriptive statistics.

	Mean (SE) or n (%)	95% CI
Background factors:		
Maternal menarcheal age	12.7 (0.065)	12.601, 12.857
Black	83 (12.7%)	-
Latina	40 (6.1%)	-
Asian/PI	11 (1.7%)	-
‘Other’	22 (3.3%)	-
White	499 (76.2%)	-
Child health:		
General health rating (coded 1–4)	3.4 (0.027)	3.323, 3.427
BMI percentile	60.6 (1.167)	58.342, 62.921
BMI percentile ≥85^th^	157 (23.9%)	-
Child adversity:		
Mother, college degree+	240 (36.6%)	-
Father, college degree+	252 (38.5%)	-
Families, below poverty line	89 (13.6%)	-
Attachment, proportion of times secure	0.6 (0.012)	0.563, 0.611
Negative life events (coded 0–3)	1.0 (0.033)	0.956, 1.084
Pubertal timing indicators:		
Breast development (TS II)	9.8 (0.051)	9.659, 9.863
Pubic hair development (TS II)	10.2 (0.042)	10.123, 10.291
Menarcheal age	12.4 (0.052)	12.299, 12.504
Adulthood cardiometabolic risk indicators:		
WC (cm)	88.9 (0.859)	87.242, 90.635
WC risk (≥80 cm)	424 (64.7%)	-
SBP (mm Hg)	109.9 (0.638)	108.609, 111.136
DBP (mm Hg)	72.8 (0.612)	71.576, 74.005
BP risk (SBP ≥130 or DBP ≥80)	168 (25.6%)	-
HbA1c (%)	5.4 (0.127)	5.133, 5.637
HbA1c risk (≥5.7%)	270 (41.2%)	-
CRP (mg/L)	5.4 (0.282)	4.873, 5.994
CRP risk (≥10 mg/L)	106 (16.2%)	-
HDL (mg/dL)	57.9 (0.751)	56.407, 59.383
HDL risk (<50 mg/dL)	182 (27.8%)	-

Means, SEs, 95% CIs or n (%) are reported from multiply imputed data for 655 participants.

### Correlations

In Table 2, correlations are reported for the prepubertal risk factors, pubertal timing indicators, and the adulthood CMR composite. As expected, significant correlations were observed among the pubertal timing indicators (all *p*s < .0001). In addition, each pubertal timing indicator was significantly correlated with the adulthood CMR composite (all *p*s < .001). With respect to the prepubertal risk factors, Black race (vs. all others) was significantly correlated with earlier breast development onset (*r* = -.162, *p* < .001), pubic hair development onset (*r* = -.232, *p* < .0001), and menarche (*r* = -.191, *p* < .0001) as well as an increase in adulthood cardiometabolic risk (*r* = .133, *p* < .01), whereas White race (vs. all others) was significantly correlated with later breast development onset (*r* = .157, *p* < .001), pubic hair development onset (*r* = .160, *p* < .001), and menarche (*r* = .213, *p* < .0001) but was not associated with adulthood cardiometabolic risk (*p*>.05). Older maternal menarcheal age was significantly correlated with later onset for all three pubertal timing indicators (all *p*s < .001). Higher BMI percentile was significantly correlated with earlier breast development onset (*r* = -.162, *p* < .01) and menarche (*r* = -.125, *p* < .05) as well as an increase in adulthood cardiometabolic risk (*r* = .209, *p* < .001), and, conversely, higher child SES was significantly correlated with later breast development onset (*r* = .121, *p* < .01) and menarche (*r* = .226, *p* < .0001) as well as a decrease in adulthood cardiometabolic risk (*r* = -.244, *p* < .0001). Finally, maternal sensitivity was significantly correlated with later menarche (*r* = .184, *p* < .0001) as well as a decrease in adulthood cardiometabolic risk (*r* = -.229, *p* < .0001). The other race and ethnicity categories, the general health rating, and the adversity exposures related to mother-child attachment and negative life events were not significantly correlated with the pubertal timing indicators or the adulthood CMR composite (all *p*s>.05).

**Table 2:** Correlations among the prepubertal risk factors, pubertal timing indicators, and adulthood CMR composite.

	Maternal menarcheal age	General health rating	BMI percentile	Child SES	Mother-child Attachment	Maternal sensitivity	Negative life events	Breast TS II	Pubic hair TS II	Menarcheal age	CMR composite
Black (vs. all other)	-.092*	-.104*	-.005	-.301****	-.142***	-.432****	-.117**	-.162***	-.232****	-.191****	.133**
Latina (vs. all other)	-.044	-,075^†^	.008	-.137***	-.077^†^	-.059	-.018	-.029	-.012	-.077	-.002
Asian/PI (vs. all other)	.019	-.062	-.062	.058	.069^†^	-.001	-.025	-.057	.009	-.050	-.061
Other (vs. all other)	-.002	-.073^†^	.027	-.081*	-.025	-.100*	-.027	.008	.060	-.012	-.019
White (vs. all other)	.092*	.174***	.007	.329****	.144***	.413****	.121**	.157***	.160***	.213****	-.077
Maternal menarcheal age	-	.043	-.012	.043	.059	.079	-.072	.176***	.207****	.369****	-.031
General health rating		-	-.118**	.167***	.043	.122**	.010	.034	-.004	.058	-.053
BMI percentile			-	-.056	-.079^†^	-.088*	.044	-.162**	-.069	-.125*	.209***
Child SES				-	.194****	.554****	.005	.121**	.066	.226****	-.244****
Mother-child attachment					-	.285****	.025	.013	.048	.080^†^	-.701
Maternal sensitivity						-	.123**	.071	.083^†^	.184****	-.229****
Negative life events							-	.050	.007	-.028	.009
Breast TS II								-	.564****	.536****	-.201****
Pubic hair TS II									-	.506****	-.180***
Menarcheal age										-	-.219***
CMR composite											-

^†^p < .10

*p < .05

**p < .01

***p < .001

****p < .0001.

### Multivariate analyses

**Fig 1:**
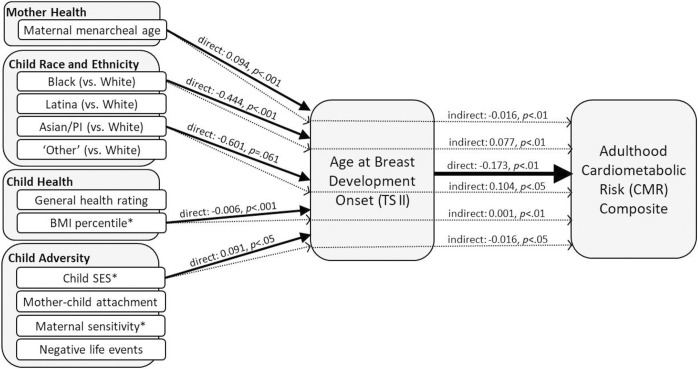
Reported from adjusted models, the solid arrows represent direct effects (with *p*-values < .10) between the prepubertal risk factors and age at breast development onset (TS II) as well as age at breast development onset (TS II) and the adulthood CMR composite. The dotted arrows represent the indirect (mediated) effects of these prepubertal risk factors on the adulthood CMR composite via age at breast development onset (TS II). Results suggest earlier pubertal onset, marked by the initiation of breast development, is a pathway through which risk for poor cardiometabolic health in adulthood is transmitted both directly and indirectly. *Direct effects (not depicted) were also observed for child BMI percentile (0.006, *p* < .01), childhood SES (-0.153, *p* < .05), and maternal sensitivity (-0.131, *p* = .080) predicting the adulthood CMR composite.

**Fig 2:**
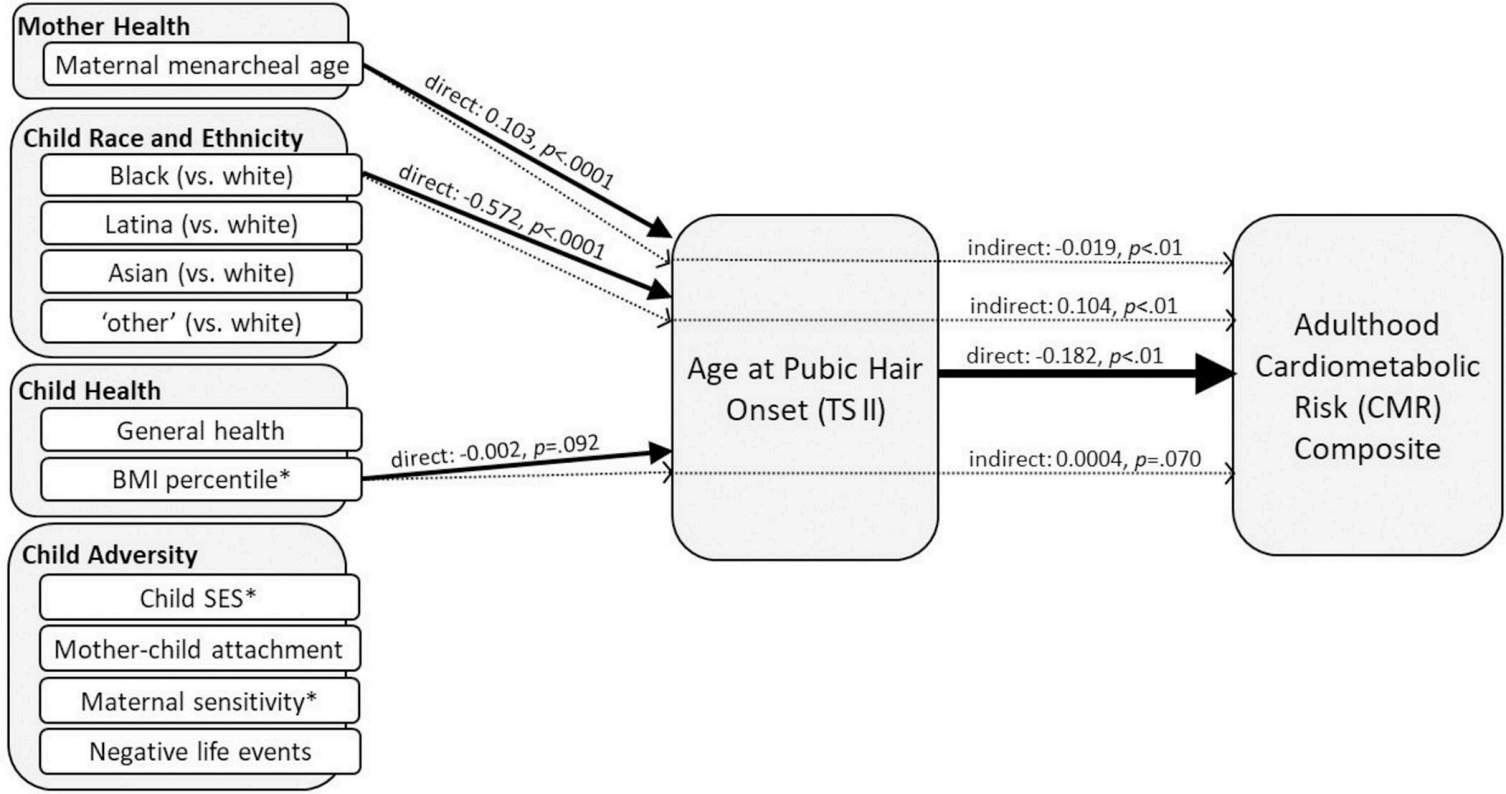
Reported from adjusted models, the solid arrows represent direct effects (with p-values < .10) between the prepubertal risk factors and age at pubic hair onset (TS II) as well as age at pubic hair onset (TS II) and the adulthood CMR composite. The dotted arrows represent the indirect (mediated) effects of these prepubertal risk factors on the adulthood CMR composite via age at pubic hair onset (TS II). Results suggest earlier pubertal onset, marked by the initiation of pubic hair, is a pathway through which risk for poor cardiometabolic health in adulthood is transmitted both directly and indirectly. *Direct effects (not depicted) were also observed for child BMI percentile (0.007, *p* < .001), child SES (-0.167, *p* < .05), and maternal sensitivity (-0.126, *p* = .074) predicting the adulthood CMR composite.

**Fig 3:**
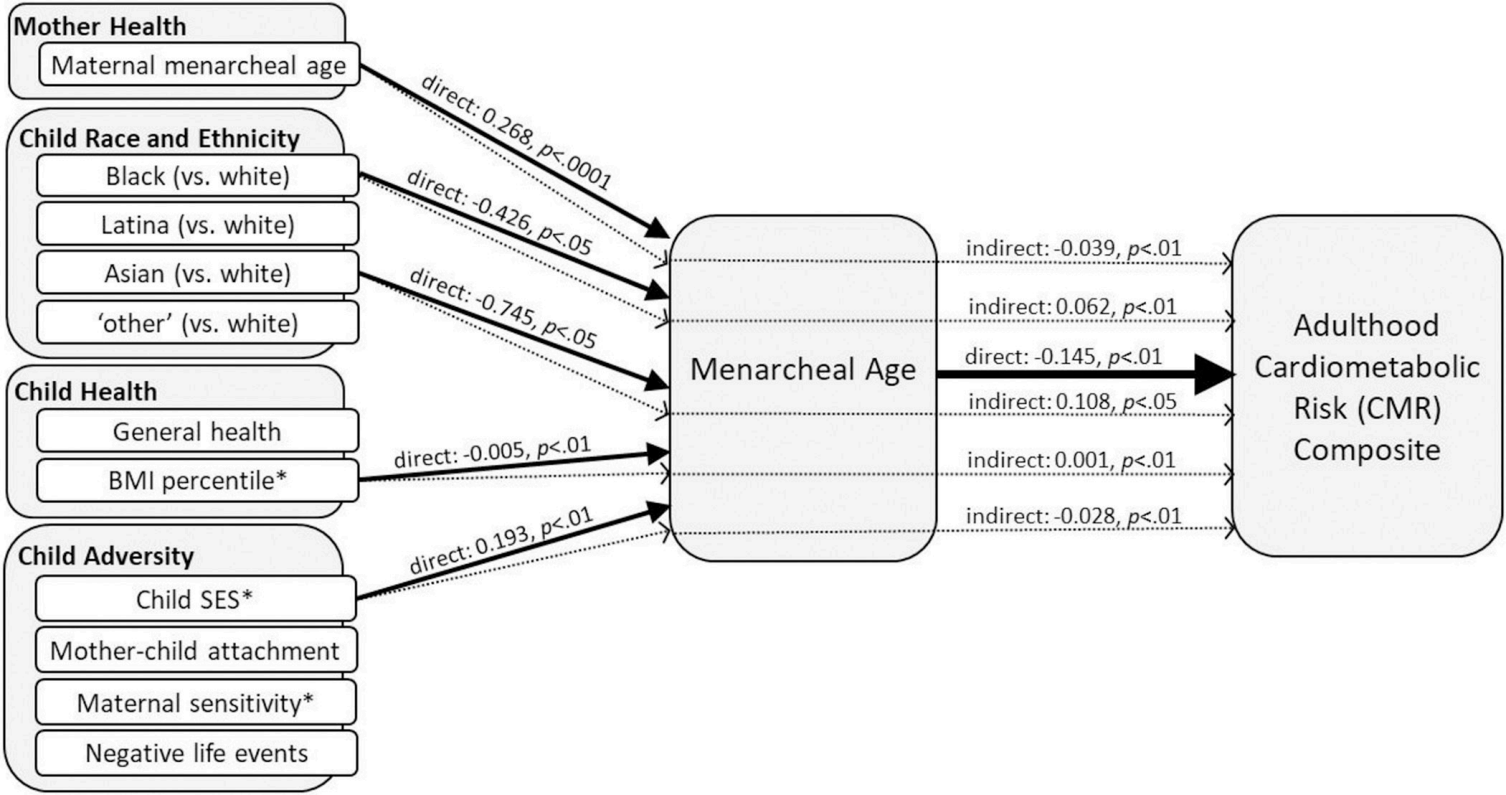
Reported from adjusted models, the solid arrows represent direct effects (with p-values < .10) between the prepubertal risk factors and menarcheal age as well as menarcheal age and the adulthood CMR composite. The dotted arrows represent the indirect (mediated) effects of these prepubertal risk factors on the adulthood CMR composite via menarcheal age. Results suggest earlier pubertal onset, marked by menarcheal age, is a pathway through which risk for poor cardiometabolic health in adulthood is transmitted both directly and indirectly. *Direct effects (not depicted) were also observed for child BMI percentile (0.006, *p* < .01) and child SES (-0.141, *p* = .076) predicting the adulthood CMR composite.

Breast development. In Table 3 and Fig 1, results are reported showing direct effects of prepubertal risk factors on age at breast development onset (TS II); direct effects of prepubertal risk factors and age at breast development onset (TS II) on the adulthood CMR composite; and mediated effects of the prepubertal risk factors on the adulthood CMR composite via age at breast development onset (TS II). Statistical significance was set to α = .05, however, all associations at *p* < .10 are described here and depicted in Figs 1–3. In the first set of analyses, older maternal menarcheal age and higher child SES predicted later breast development onset (*p* < .001, *p* < .05, respectively), whereas Black (vs. White) and Asian/PI (vs. White) identification and higher BMI percentile predicted earlier breast development onset (*p* < .001, *p* < .10, *p* < .001, respectively). In the second set of analyses, later breast development onset predicted lower adulthood CMR (*p* < .01). In addition, higher child SES and higher maternal sensitivity predicted lower adulthood CMR (*p* < .05, *p* < .10, respectively), whereas higher BMI percentile predicted higher adulthood CMR (*p* < .01). In the final set of mediation analyses, effects of maternal menarcheal age, Black (vs. White), Asian/PI (vs. White), BMI percentile, and child SES on adulthood CMR were all significantly mediated by the timing of breast development onset (all *p*s < .05).

**Table 3:** Adjusted coefficients examining direct effects of prepubertal risk factors on age at breast development onset (TS II); direct effects of prepubertal risk factors and age at breast development onset (TS II) on the adulthood CMR composite; and mediated effects of prepubertal risk factors on the adulthood CMR composite via age at breast development onset (TS II).

	Prepubertal risk factors, pubertal timing (breast), and adulthood cardiometabolic risk					
	Direct Effects: Prepubertal risk factors → pubertal timing (breast TS II)		Direct Effects: Prepubertal risk factors and pubertal timing (breast TS II) → CMR composite		Mediated Effects: Prepubertal risk factors → pubertal timing (breast TS II) → CMR composite	
	DV: Breast TS II		DV: CMR Composite		DV: CMR Composite	
	Effect Estimate	95% CI	Effect Estimate	95% CI	Effect Estimate	95% CI
**PREDICTORS:**						
Prepubertal risk factors:						
Maternal menarcheal age	0.094***	0.043, 0.145	0.010	-0.063, 0.082	-0.016**	-0.032, -0.005
Black (vs. White)	-0.444***	-0.679, -0.210	0.001	-0.302, 0.305	0.077**	0.023, 0.148
Latina (vs. White)	-0.130	-0.456, 0.196	-0.161	-0.637, 0.315	-	-
Asian/PI (vs. White)	-0.601^†^	-1.230, 0.028	-0.420	-1.278, 0.437	0.104*	-0.004, 0.253
Other (vs. White)	-0.028	-0.455, 0.398	-0.278	-0.836, 0.279	-	-
General health rating	-0.035	-0.169, 0.098	0.002	-0.150, 0.154	-	-
BMI percentile	-0.006***	-0.008, -0.003	0.006**	0.002, 0.010	0.001**	0.0003, 0.002
Child SES	0.091*	0.002, 0.179	-0.153*	-0.304, -0.001	-0.016*	-0.038, 0.0001
Mother-child attachment	-0.080	-0.358, 0.198	0.030	-0.351, 0.411	-	-
Maternal sensitivity	-0.064	-0.163, 0.036	-0.131^†^	-0.278, 0.016	-	-
Negative life events	-0.051	-0.150, 0.048	0.011	-0.149, 0.170	-	-
Pubertal timing indicator:						
Breast TS II	-	-	-0.173**	-0.279, -0.068	-	-

^†^p < .10

*p< .05

**p< .01

***p< .001

****p< .0001.

Note: For the indirect effects of Asian/PI and Child SES, there were discrepancies between the corresponding *p*-values (both *p* < .05) and 95% CIs (both suggesting *p*>.05). In both cases, the joint test suggested the indirect effect had a *p*-value close to .05 and, in those cases, the partial posterior (for exact *p*-values) and empirical Bayes (for CIs) methods demonstrated some variation of estimation around the .05 threshold.

Pubic hair development. In Table 4 and Fig 2, results are reported showing direct effects of prepubertal risk factors on age at pubic hair onset (TS II); direct effects of prepubertal risk factors and age at pubic hair onset (TS II) on the adulthood CMR composite; and mediated effects of the prepubertal risk factors on the adulthood CMR composite via age at pubic hair onset (TS II). In the first set of analyses, older maternal menarcheal age predicted later pubic hair onset (*p* < .0001), while Black (vs. White) and higher BMI percentile predicted earlier pubic hair onset (*p* < .0001, p < .10, respectively). In the second set of analyses, later pubic hair onset predicted lower adulthood CMR (*p* < .01). In addition, higher child SES and higher maternal sensitivity predicted lower adulthood CMR (*p* < .05, *p* < .10, respectively), while higher BMI percentile predicted higher adulthood CMR (*p* < .001). In the final set of mediation analyses, effects of maternal menarcheal age and Black (vs. White) on adulthood CMR were significantly mediated by the timing of pubic hair onset (all *p*s < .05), and the effect of BMI percentile on adulthood CMR was mediated by the timing of pubic hair onset at the level of a trend (*p* = .07).

**Table 4:** Adjusted coefficients examining direct effects of prepubertal risk factors on age at pubic hair onset (TS II); direct effects of prepubertal risk factors and age at pubic hair onset (TS II) on the adulthood CMR composite; and mediated effects of prepubertal risk factors on the adulthood CMR composite via age at pubic hair onset (TS II).

	Prepubertal risk factors, pubertal timing (pubic hair), and adulthood cardiometabolic risk					
	Direct Effects: Prepubertal risk factors → pubertal timing (pubic hair TS II)		Direct Effects: Prepubertal risk factors and pubertal timing (pubic hair TS II) → CMR composite		Mediated Effects: Prepubertal risk factors → pubertal timing (pubic hair TS II) → CMR composite	
	DV: Pubic Hair TS II		DV: CMR Composite		DV: CMR Composite	
	Effect Estimate	95% CI	Effect Estimate	95% CI	Effect Estimate	95% CI
**PREDICTORS:**						
Prepubertal risk factors:						
Maternal menarcheal age	0.103****	0.055, 0.151	0.012	-0.061, 0.085	-0.019**	-0.036, -0.005
Black (vs. White)	-0.572****	-0.797, -0.348	-0.027	-0.338, 0.284	0.104**	0.032, 0.194
Latina (vs. White)	-0.096	-0.438, 0.246	-0.156	-0.632, 0.320	-	-
Asian/PI (vs. White)	-0.093	-0.715, 0.528	-0.334	-1.196, 0.528	-	-
Other (vs. White)	0.175	-0.234, 0.584	-0.242	-0.790, 0.306	-	-
General health rating	-0.056	-0.169, 0.057	-0.002	-0.156, 0.152	-	-
BMI percentile	-0.002^†^	-0.005, 0.0004	0.007***	0.003, 0.010	0.0004^†^	-0.000, 0.001
Child SES	0.010	-0.079, 0.099	-0.167*	-0.315, -0.019	-	-
Mother-child attachment	0.025	-0.221, 0.271	0.048	-0.330, 0.426	-	-
Maternal sensitivity	-0.035	-0.129, 0.060	-0.126^†^	-0.274, 0.021	-	-
Negative life events	0.002	-0.101, 0.105	0.020	-0.141, 0.180	-	-
Pubertal timing indictor:						
Pubic hair TS II	-	-	-0.182**	-0.303, -0.062	-	-

^†^p < .10

*p< .05

**p< .01

***p< .001

****p< .0001.

Menarcheal age. In Table 5 and Fig 3, results are reported showing direct effects of prepubertal risk factors on menarcheal age; direct effects of prepubertal risk factors and menarcheal age on the adulthood CMR composite; and mediated effects of the prepubertal risk factors on the adulthood CMR composite via menarcheal age. In the first set of analyses, older maternal menarcheal age and higher child SES predicted later menarche (*p* < .0001, *p* < .01, respectively), while Black (vs. White) and Asian/PI (vs. White) identification and higher BMI percentile predicted earlier menarche (*p* < .05, *p* < .05, *p* < .01, respectively). In the second set of analyses, later menarche predicted lower adulthood CMR (*p* < .01). In addition, higher child SES predicted lower adulthood CMR (*p* < .10), while higher BMI percentile predicted higher adulthood CMR (*p* < .01). In the final set of mediation analyses, effects of maternal menarcheal age, Black (vs. White), Asian/PI (vs. White), BMI percentile, and child SES on adulthood CMR were all significantly mediated by the timing of menarche (all *p*s < .05).

**Table 5:** Adjusted coefficients examining direct effects of prepubertal risk factors on menarcheal age; direct effects of prepubertal risk factors and menarcheal age on the adulthood CMR composite; and mediated effects of prepubertal risk factors on the adulthood CMR composite via menarcheal age.

	Prepubertal risk factors, pubertal timing (menarcheal age), and adulthood cardiometabolic risk					
	Direct Effects: Prepubertal risk factors → pubertal timing (menarcheal age)		Direct Effects: Prepubertal risk factors and pubertal timing (menarcheal age) → CMR composite		Mediated Effects: Prepubertal risk factors → pubertal timing (menarcheal age) → CMR composite	
	DV: Menarcheal Age		DV: CMR Composite		DV: CMR Composite	
	Effect Estimate	95% CI	Effect Estimate	95% CI	Effect Estimate	95% CI
**PREDICTORS:**						
Prepubertal risk factors:						
Maternal menarcheal age	0.268****	0.208, 0.328	0.032	-0.042, 0.106	-0.039**	-0.067, -0.013
Black (vs. White)	-0.426*	-0.754, -0.098	0.016	-0.289, 0.321	0.062**	0.009, 0.136
Latina (vs. White)	-0.263	-0.668, 0.141	-0.176	-0.655, 0.303	-	-
Asian/PI (vs. White)	-0.745*	-1.412, -0.079	-0.425	-1.277, 0.427	0.108*	0.007, 0.251
Other (vs. White)	-0.061	-0.590, 0.467	-0.282	-0.833, 0.269	-	-
General health rating	-0.040	-0.186, 0.107	0.002	-0.151, 0.156	-	-
BMI percentile	-0.005**	-0.009, -0.001	0.006**	0.002, 0.010	0.001**	0.0001, 0.002
Child SES	0.193**	0.076, 0.310	-0.141^†^	-0.296, 0.015	-0.028**	-0.057, -0.007
Mother-child attachment	0.010	-0.325, 0.346	0.046	-0.336, 0.428	-	-
Maternal sensitivity	-0.001	-0.126, 0.125	-0.120	-0.269, 0.028	-	-
Negative life events	-0.024	-0.151, 0.103	0.016	-0.143, 0.175	-	-
Pubertal timing indicator:						
Menarcheal age	-	-	-0.145**	-0.239, -0.051	-	-

^†^p < .10

*p< .05

**p< .01

***p< .001

****p< .0001.

## Discussion

There is a strong and growing focus on understanding how exposures in childhood may shape risk for poor health and disease over the life course. These efforts, however, have been limited with respect to considering the critical role of pubertal development and its timing. Puberty may play a key role in transmitting early risk as its timing is influenced by prepubertal risk factors (e.g., child health and adversity), and its timing, in turn, predicts a range of maladaptive psychosocial and health outcomes (e.g., depression, cardiometabolic disease), even into adulthood. The current study was unique as it possessed data that traversed these developmental periods, allowing the integrated examination of links between prepubertal risk factors, pubertal timing, and adulthood cardiometabolic risks, modeling, specifically, the potential mediating role of pubertal timing as a pathway through which early risk may influence adulthood cardiometabolic health.

In the current study, a consistent pattern of results emerged, providing support for the mediational role of pubertal timing. First, among the examined prepubertal risk factors, younger maternal menarcheal age, Black or Asian/PI race/ethnicity (vs. White), higher child BMI percentile, and lower child SES predicted earlier breast development onset (TS II) and younger menarcheal age, both markers of gonadarche. In parallel, prepubertal risk factors, younger maternal menarcheal age, Black race/ethnicity (vs. White), and higher child BMI percentile, predicted earlier pubic hair onset (TS II), a marker of adrenarche. Each of these predictors is well-established in the literature except for Asian/PI race/ethnicity, which will require future examination in samples with a larger number of Asian girls. Second, all three pubertal timing indicators, earlier breast development onset (TS II), earlier pubic hair onset (TS II), and younger menarcheal age, predicted poorer cardiometabolic health in adulthood, as indexed by the adulthood CMR composite. Finally, the examination of indirect effects showed all three pubertal timing indicators mediated effects of the prepubertal risk factors on cardiometabolic health in adulthood. That is, the timing of breast development onset (TS II) and menarcheal age partially transmitted effects of prepubertal risk factors (maternal menarcheal age, child race/ethnicity, child BMI percentile, and child SES) on the adulthood CMR composite, while the timing of pubic hair onset (TS II) partially transmitted effects of prepubertal risk factors (maternal menarcheal age, child race/ethnicity, and child BMI percentile) on the adulthood CMR composite.

The clinical implications of the current finding that pubertal timing may play a larger role as a pathway linking early life exposures to cardiometabolic health in adulthood are far-reaching. Although it is already known that earlier developing girls are at risk for a host of poor outcomes (e.g., depression, poor self-image, poor academic performance, obesity, type 2 diabetes, incident cardiovascular disease, early mortality) [30–43], understanding the role of pubertal development as a pathway raises novel opportunities for early surveillance and intervention. For example, the identification of prepubertal risk factors related to younger maternal menarcheal age and minority race/ethnic status may offer opportunities for additional surveillance to offset potential harms associated with earlier pubertal onset. Whereas the identification of other prepubertal risk factors that are modifiable such as prepubertal obesity and child SES may offer novel opportunities for intervention to slow the onset of puberty, thereby preventing harms associated with earlier pubertal onset. In both cases, results from the current study suggest that leveraging knowledge associated with pubertal timing may aid early surveillance or intervention efforts that have implications for the preservation of health and well-being into adulthood.

With respect to modifiable prepubertal risk factors specifically, evidence suggests that higher prepubertal BMI, examined both continuously and in categories of overweight and obese, is a strong predictor of earlier pubertal onset [22,66–71], accounting for the largest proportion of variance in pubertal onset compared to other predictors [72]. In addition, trajectories of accelerated weight gain in infancy and early childhood also predict earlier pubertal onset. [23,73–77]. Building on this foundation, two studies have conducted interventions to slow pubertal onset. In one study, a multifaceted lifestyle intervention was performed among overweight/obese girls in the prepubertal period. [78]. Results showed that girls who exhibited weight loss (vs. no weight change or increase) were less likely to experience pubertal onset at the 1-year follow-up. In a second study, a multifaceted school-based intervention (vs. control) was performed to prevent obesity [79]. Results showed that girls who attended intervention schools were less likely to experience menarche at the 2-year follow-up. These effects were mediated by changes in the intervention targets, including lower weight and body fat increases, reduced screen time, and increased physical activity. Taken together, these findings are intriguing in pointing to the possibility that pubertal onset can be manipulated through lifestyle-based intervention, providing support for the targeting of pubertal development as a means to improve health more broadly.

A notable strength of the current study was the use of data from the landmark NICHD SECCYD and recent SECCYD follow-up study, SHINE. This longitudinal study of more than 30 years offered the unique opportunity to test a series of life course models using real-time, state-of-the-art methods and measures. Rigorous measurement of the prepubertal risk factors included assessment of child health and adversity exposures using, for example, repeated assessments of videotaped mother–child interactions in well-validated study tasks coded to assess mother–child attachment patterns and maternal sensitivity as well as real-time mother reports of socioeconomic conditions and early life stressors. The pubertal timing assessments were similarly comprehensive. Tanner staging was derived from annual physical exams (with breast bud palpation) performed by trained medical providers to assess dimensions of breast development and pubic hair growth as well as real-time self-reports to assess age at menarche. Finally, the adulthood CMR composite represented key components of adulthood cardiometabolic health measured according to best practices in areas of anthropometrics, blood pressure, and blood collection, processing, and analysis. A notable weakness of the current study was the small number of non-White girls, limiting the examination of individual racial/ethnic groups and the generalizability of the results. In addition, the current study lacked assessment of key prepubertal health variables. For example, pre-pubertal blood samples were not obtained which would have enabled the examination of relevant underlying biological processes such as insulin resistance.

Future research should address the weaknesses of the current study. Most importantly, samples should include a larger number of girls from minority racial and ethnic backgrounds to further elaborate on the current model, focusing specifically on better understanding racial and ethnic disparities in pubertal timing and cardiometabolic health outcomes. Consistent with findings in the current study, it is well established that Black girls in particular experience puberty earlier [17–19]; compared with White girls, their breast development onset, pubic hair onset, and menarche occurred 9.6 months, 12 months, and 7.2 months earlier, respectively, in a prior study [20]. Also consistent with the current study, these associations were independent of BMI and SES [20], underscoring that more work is needed to identify factors accounting for these differences. Such work would be informed by incorporating adversity exposures that are unique to minority girls, such as institutional racism and personal experiences of discrimination and bias. Relatedly, more work is needed to breakdown the impacts of SES-related exposures, which disproportionately burden minority families [80,81] and may intersect with other health-damaging stressors in vulnerable communities [82–84]. Future research should expand on the measurement of early life health behaviors and health status indicators to help elucidate the biological linkages between the prepubertal risk factors, pubertal timing indicators, and adulthood cardiometabolic health. Finally, it is also worth noting that the results should be considered preliminary with additional investigation needed both to replicate findings and to better characterize the clinical meaning of the links observed in the current study.

In summary, findings in the current study contribute to the broader literature by identifying pubertal development and its timing as a potentially important pathway through which early life exposures may shape adulthood cardiometabolic health and disease. These findings have important implications for novel opportunities for increased surveillance and potential intervention focusing on pubertal development as a target to improve health more broadly.
